# A Novel Two-Step Hierarchical Quantitative Structure–Activity Relationship Modeling Work Flow for Predicting Acute Toxicity of Chemicals in Rodents

**DOI:** 10.1289/ehp.0800471

**Published:** 2009-04-03

**Authors:** Hao Zhu, Lin Ye, Ann Richard, Alexander Golbraikh, Fred A. Wright, Ivan Rusyn, Alexander Tropsha

**Affiliations:** 1 Laboratory for Molecular Modeling, Division of Medicinal Chemistry and Natural Products, School of Pharmacy, University of North Carolina at Chapel Hill, Chapel Hill, North Carolina, USA; 2 National Center for Computational Toxicology, Office of Research and Development, U.S. Environmental Protection Agency, Research Triangle Park, North Carolina, USA; 3 Department of Biostatistics and; 4 Department of Environmental Sciences and Engineering, School of Public Health, University of North Carolina at Chapel Hill, Chapel Hill, North Carolina, USA

**Keywords:** acute toxicity, computational toxicology, IC_50_, LD_50_, LOAEL, NOAEL, QSAR

## Abstract

**Background:**

Accurate prediction of *in vivo* toxicity from *in vitro* testing is a challenging problem. Large public–private consortia have been formed with the goal of improving chemical safety assessment by the means of high-throughput screening.

**Objective:**

A wealth of available biological data requires new computational approaches to link chemical structure, *in vitro* data, and potential adverse health effects.

**Methods and results:**

A database containing experimental cytotoxicity values for *in vitro* half-maximal inhibitory concentration (IC_50_) and *in vivo* rodent median lethal dose (LD_50_) for more than 300 chemicals was compiled by Zentralstelle zur Erfassung und Bewertung von Ersatz- und Ergaenzungsmethoden zum Tierversuch (ZEBET; National Center for Documentation and Evaluation of Alternative Methods to Animal Experiments). The application of conventional quantitative structure–activity relationship (QSAR) modeling approaches to predict mouse or rat acute LD_50_ values from chemical descriptors of ZEBET compounds yielded no statistically significant models. The analysis of these data showed no significant correlation between IC_50_ and LD_50_. However, a linear IC_50_ versus LD_50_ correlation could be established for a fraction of compounds. To capitalize on this observation, we developed a novel two-step modeling approach as follows. First, all chemicals are partitioned into two groups based on the relationship between IC_50_ and LD_50_ values: One group comprises compounds with linear IC_50_ versus LD_50_ relationships, and another group comprises the remaining compounds. Second, we built conventional binary classification QSAR models to predict the group affiliation based on chemical descriptors only. Third, we developed *k*-nearest neighbor continuous QSAR models for each subclass to predict LD_50_ values from chemical descriptors. All models were extensively validated using special protocols.

**Conclusions:**

The novelty of this modeling approach is that it uses the relationships between *in vivo* and *in vitro* data only to inform the initial construction of the hierarchical two-step QSAR models. Models resulting from this approach employ chemical descriptors only for external prediction of acute rodent toxicity.

Development of accurate and predictive *in vitro* toxicity testing methods that could be used as alternatives for lengthy and costly *in vivo* experiments has long been an elusive goal for both industry and regulatory agencies (National Research Council 2007). New, bold research programs were recently established at the National Toxicology Program (Xia et al. 2008) and the U.S. Environmental Protection Agency (U.S. EPA) (Dix et al. 2007) and coordinated at the interagency level by the U.S. government (Collins et al. 2008) to address this important challenge in a systematic way. The overall goal of these initiatives is to explore a diverse array of *in vitro* toxicity assays, such as cell-based and cell-free high-throughput screening (HTS) techniques, as well as toxicogenomic technologies, to evaluate the toxic potential of chemicals and prioritize candidates for animal testing. However, the utility of *in vitro* data as indicators of *in vivo* effects will be fully realized only if rigorous correlation between the toxicity of chemicals *in vitro* and *in vivo* can be established (National Research Council 2007; Rabinowitz et al. 2008).

Many previous studies have indicated that the correlation between the *in vitro* toxicity results and animal toxicity test data (e.g., acute, subacute, subchronic, and chronic rodent toxicity test results) is generally poor. Most notably, in 2001, the Interagency Coordinating Committee on the Validation of Alternative Methods (ICCVAM) hosted a workshop to assess the relationship between cytotoxicity and rodent acute toxicity for > 300 diverse compounds; the data were compiled by the Zentralstelle zur Erfassung und Bewertung von Ersatz-und Ergaenzungsmethoden zum Tierversuch (ZEBET; the National Center for Documentation and Evaluation of Alternative Methods to Animal Experiments) [ICCVAM and National Toxicology Program Interagency Center for the Evaluation of Alternative Toxicological Methods (NICEATM) 2001]. It was concluded that there is no clear correlation between cytotoxicity [half-maximal inhibitory concentration (IC_50_)] and acute toxicity [median lethal dose (LD_50_)] data in rodents. Similarly, poor correlation was found between *in vitro* cytotoxicity and *in vivo* rodent carcinogenicity, even when a diverse set of *in vitro* end points from HTS was used (Xia et al. 2008; Zhu et al. 2008).

Cheminformatics approaches such as quantitative structure–activity relationship (QSAR) modeling have been widely used in toxicology (Dearden 2003; Johnson et al. 2004). Several software packages, such as Toxicity Prediction by Komputer Assisted Technology (TOPKAT) (Venkatapathy et al. 2004) and Multiple Computer-Automated Structure Evaluation (MultiCASE) (Matthews et al. 2006), have been developed and actively used by both industry and regulatory agencies. However, existing modeling tools generally do not achieve good external accuracy of prediction for compounds not used in model development, and few QSAR models have been successful in predicting *in vivo* toxicity end points for diverse sets of environmental compounds (Benigni et al. 2007; Stouch et al. 2003).

There are several possible reasons that previous attempts to establish relationships between *in vitro* and *in vivo* toxicity data were largely ineffective. These include, among other factors, inadequate attention paid to the chemical diversity of the compounds used for screening and modeling and, consequently, unjustified confidence in the ability of models to extrapolate significantly outside the chemistry space of the training set. Furthermore, the conventional QSAR modeling efforts have been disconnected from the growing efforts to employ *in vitro* screening (i.e., HTS data) to predict *in vivo* outcomes. Recently, we have proposed the use of hybrid chemical–biological descriptors, that is, a combination of conventional chemical descriptors with HTS profile data regarded as biological descriptors. We have demonstrated that these hybrid descriptors afford QSAR models with significantly higher accuracy of prediction of rodent carcinogenicity versus models using chemical descriptors alone, and much higher accuracy versus models that used biological *in vitro* data alone (Zhu et al. 2008).

These recent studies suggest that the explicit consideration of chemical structure (in the form of chemical descriptors) along with *in vitro* assay data could potentially account for discrepancies between *in vitro* and *in vivo* results and produce more accurate predictive models of *in vivo* toxicity. To validate this hypothesis further, in this study we used the ZEBET data set (ICCVAM and NICEATM 2001) for which previous attempts to establish the direct *in vitro*/*in vivo* correlation proved largely unsuccessful (Freidig et al. 2007). We have observed that chemicals can be partitioned into two classes based on comparison between cytotoxicity and acute toxicity data: *a*) those for which the linear *in vitro* (IC_50_)/*in vivo* (LD_50_) correlation could be demonstrated and *b*) those that correlate poorly. Furthermore, and of central importance for applying our models to the external set of chemicals for which no *in vitro* data exist, we have built binary QSAR models that could discriminate between compounds in these two classes with reasonable accuracy based on their chemical features alone. Finally, we have established rigorous and externally predictive class-specific QSAR models of rodent acute toxicity measured by LD_50_ values. We show that a two-step hierarchical QSAR modeling work flow where compounds are first assigned to a class using binary QSAR models and then their LD_50_ values is predicted using class-specific continuous QSAR models affords accurate prediction of LD_50_ values for compounds not included in the training set. In addition, we show that this two-step model’s statistical prediction accuracy compares favorably with currently available commercial toxicity predictors. Our studies suggest that the two-step QSAR modeling work flow can improve performance of predictive acute toxicity models for diverse organic compounds and aid in prioritizing compounds for rodent toxicity testing.

## Materials and Methods

### Data sets

The ZEBET database consists of data for 361 chemicals compiled from literature studies and published in a consolidated ICCVAM report (ICCVAM and NICEATM 2001). Every compound in this data set has at least one cytotoxicity result (IC_50_) and at least one type of rodent acute toxicity value (rat or mouse LD_50_). We defined ZEBET criteria to select cytotoxicity data for this data set as follows: *a*) at least two different IC_50_ values were available, either from different cell types or from different cytotoxicity end points; *b*) cytotoxicity data were obtained with mammalian cells; *c*) cytotoxicity data obtained with hepatocytes were not acceptable; and *d*) chemical exposure time in the cytotoxicity tests was at least 16 hr. Furthermore, only the results obtained from the following cytotoxicity tests were accepted: *a*) cell proliferation measured by cell number, protein, DNA content, DNA synthesis, or colony formation; *b*) cell viability and metabolic indicators, including metabolic inhibition test (MIT-24), 3-(4,5-dimethylthiazol-2-yl)-2,5-diphenyltetrazolium bromide (MTT) assay, 3-(4,5-dimethylthiazol-2-yl)-5-(3-carboxymethoxyphenyl)-2-(4-sulfophenyl)-2H-tetrazolium (MTS) assay, and sodium 3,3-(1-[(phenylamino)carbonyl]-3,4-tetrazolium)-bis(4-emthoxy-6-nitro)benzene sulfonic acid hydrate (XTTC); *c*) cell viability and membrane indicators, including neutral red uptake, trypan blue exclusion, cell attachment, and cell detachment; and *d*) differentiation indicators.

For the purpose of this work, we curated the data set to select the subset of organic compounds and excluded inorganic and organometallic compounds, as well as compound mixtures, because conventional chemical descriptors used in QSAR studies could not be computed in these cases. There were 254 and 235 compounds that had rat or mouse LD_50_ (millimole/kilogram-body weight/day) values, respectively. Only LD_50_ values published in the Registry of Toxic Effects of Chemical Substances (RTECS) (Norager et al. 1978; Ruden and Hansson 2003) were used. The distributions of log(1/LD_50_) values of ZEBET compounds, with the exception of a single outlier, were from −2.61 to 2.30 for the rat and from −2.50 to 2.19 for the mouse. We considered one compound, 2,3,7,8-tetrachlorodibenzo-*p*-dioxin (CAS 1746-01-6), an activity outlier because its log(1/LD_50_) value was −4.21 for the rat, which deviated significantly from the activity range of the data set. After excluding this single outlier, the data sets used for modeling consisted of 253 compounds for the rat and 235 compounds for the mouse. An additional set of 115 compounds with complete data (both LD_50_ and IC_50_) for the rat was recently released by ICCVAM, which we used for validation (referred to as the ICCVAM data set). [For raw data, see Supplemental Material, Table 1 (doi:10.1289/ehp.0800471.S1).]

The data on rat chronic lowest observed adverse effect levels (LOAELs) and rat chronic no observed adverse effect levels (NOAELs) were compiled from an internal low-dose toxicity data set established in our laboratory [see Supplemental Material, Table 2 (doi:10.1289/ehp.0800471.S1)]. These data include a combination of multiple toxicity phenotypes, such as liver toxicity and kidney toxicity. Compared with the ZEBET data set, 42 unique compounds have both rat LOAEL and *in vitro* IC_50_ values, and 41 compounds have both NOAEL and *in vitro* IC_50_ values. Because of limited availability of LOAEL and NOAEL data, we used these two data sets only to illustrate the data partitioning algorithm and did not build any QSAR models for them.

### QSAR modeling approaches

We used the *k*-nearest neighbor (*k*NN) QSAR modeling approach that has been developed in our group (Zheng and Tropsha 2000). In brief, the method is based on the *k*NN principle and the variable selection procedure. It employs the leave-one-out cross-validation procedure (LOO-CV) and a simulated-annealing algorithm for the variable selection. The procedure starts with the random selection of a predefined number of descriptors from all descriptors. If *k*NN > 1, the estimated activities *ŷ_i_* of compounds excluded by the LOO procedure are calculated using the following formula:


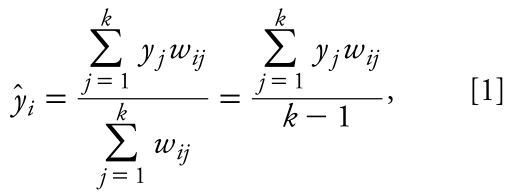


where *y_j_* is the activity of the *j*th compound. We define weights *w_ij_* as


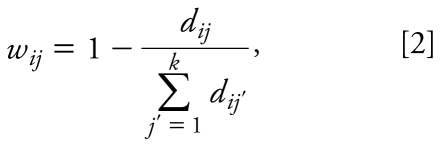


where *d_ij_* is the Euclidean distance between compound *i* and its *j*th nearest neighbor. Further details of the algorithms and work flow are provided elsewhere (Medina-Franco et al. 2005; Ng et al. 2004; Shen et al. 2002; Zheng and Tropsha 2000).

We developed rat and mouse LD_50_ QSAR models for ZEBET compounds using DRAGON chemical descriptors (DRAGON for Windows, version 5.4; Teleste s.r.l., Milan, Italy). Before model construction, 23 compounds with rat LD_50_ results and 24 compounds with mouse LD_50_ results were selected at random to serve as external validation sets. The remaining 230 rat and 211 mouse compounds were used as modeling sets, and each was divided multiple times into training/test sets using the sphere exclusion approach (Golbraikh et al. 2003). We characterized the statistical significance of the models with the standard LOO-CV *R*^2^ (*q*^2^) for the training sets and the conventional *R*^2^ for the test sets when modeling real values (i.e., continuous QSAR). For classification modeling, we used correct classification rates expressed as a fractional value between 0 and 1. The model acceptability cutoff values of the LOO-CV accuracy of the training sets and the prediction accuracy for test sets were both set to 0.65 for classification models. For continuous models, the acceptability thresholds for LOO-CV regression *q*^2^ for the training sets and *R*^2^ values for the test set were both set at 0.5. Models that did not meet both training and test set cutoff criteria were discarded.

### Moving M-regression for data partitioning

We used a novel approach related to a class of M-regression methods (Andersen 2007), which we termed “moving M-regression,” to select compounds for which there is a strong correlation between IC_50_ and LD_50_ values (class 1). The approach is a variant of the least squares regression that takes into account only those data points contained within a band around the regression line *y*^regr^ = *ax* + *b*. For each *y*, only the points within the interval [*y* − *d_i_*, *y* + *d_i_*] are candidates for class 1, whereas points outside of this band are excluded from class 1. If the line *y* = *ax* + *b* is moved, some new points will enter the band, whereas some other points will leave it, which may result in a higher regression *R*^2^; this also explains why we use the term “moving M-regression.” For each point, we define {*x_i_*, *y_i_*}, *i* = 1, …, *n*, the moving M-regression inclusion function, as


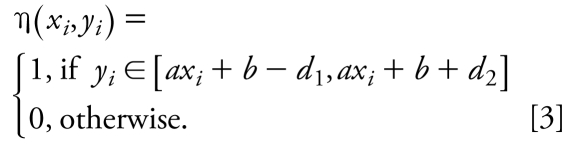


Thus, the moving M-regression line can be found by minimizing the following expression:


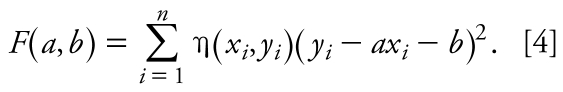


Function *F* is not differentiable at all points (*x_i_*, *y_i_*) such that *y_i_* = *ax_i_* + *b* − *d*_1_ and *y_i_* = *ax_i_* + b + *d*_2_. For practical purposes, we approximate η(*x_i_*, *y_i_*) by sums of two sigmoid functions:


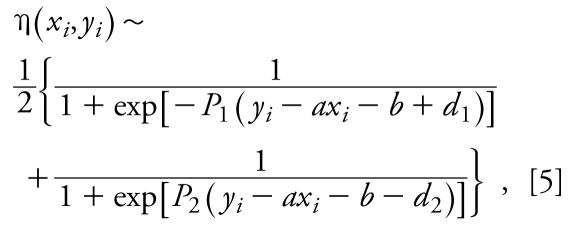


where *P*_1_ and *P*_2_ are large (~ 100) positive parameters. Indeed, as *P*_1_ and *P*_2_ approach infinity, the expression on the right side of Equation 5 approaches the right side of Equation 3. Small approximation errors in the vicinity of points {*ax_i_* + *b* − *d*_1_, *y_i_*} and {*ax_i_* + *b* + *d*_2_, *y_i_*} approach zero as both *P*_1_ and *P*_2_ approach infinity. It is as if the data points are gradually included within, or excluded from, the band when the regression line is moving. Finally, replacing η(*x_i_*, *y_i_*) by Equation 3, we obtain


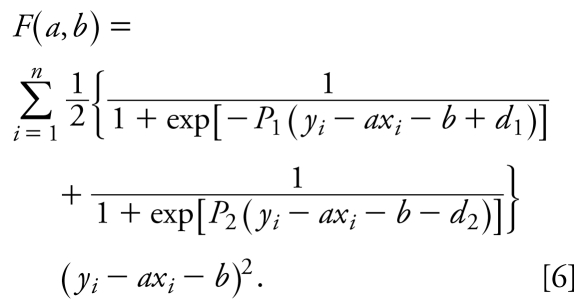


To optimize *F*(*a*, *b*), the following system of equations is to be solved:


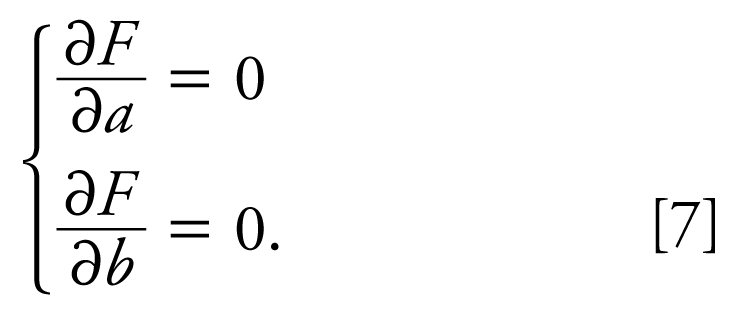


Equations 7 are nonlinear, so depending on the data set and parameters *P*_1_, *P*_2_, *d*_1_, and *d*_2_, they can have multiple solutions (*a*, *b*).

In these studies, *x_i_* and *y_i_* were the *in vitro* log(1/IC_50_) and *in vivo* log(1/LD_50_) values, respectively, for a data set of compounds under study. Instead of using Equation 6, we determined the compounds that belong to class 1 by maximizing the number of data points within the band. With this correction, our target function takes the form


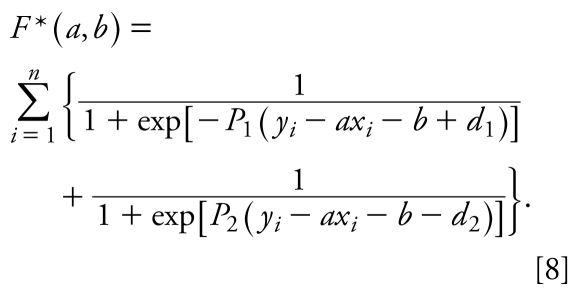


To obtain the baseline toxicity regression (see Results), we opted to minimize the number of outliers below the regression line. For this purpose, we added additional terms for the lower border of the band weighted by an arbitrary parameter α. Thus, we minimized the following target function:


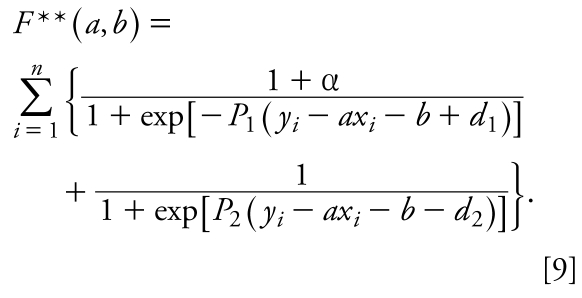


**Figure 1:**
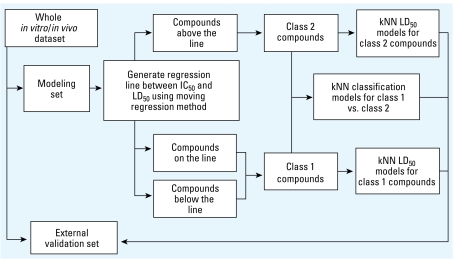
The work flow of the two-step *k*NN QSAR LD_50_ modeling.

The initial point (*a*, *b*) for minimization of F** was selected manually. *P*_1_ and *P*_2_ were equal to 100, *d*_1_ and *d*_2_ were equal to 0.4, and α was equal to 1. To optimize Equation 9, the system of Equations 7 in which *F* is replaced by F** should be solved. Figure 1 summarizes the data analytical work flow that we employed in this study for rodent acute toxicity modeling.

### Model validation

We validated training set models by evaluating their external predictive power on the test sets as described above. Furthermore, a 5-fold external CV analysis was performed for the original ZEBET data set: the data set was randomly split into five equal-size subsets of compounds and five independent sets of calculations were conducted each time using 80% of the whole data set as a modeling set and the remaining 20% compounds as a test set. In addition, robustness of QSAR models was verified using a Y-randomization (randomization of response) approach as follows. We randomly divided the modeling set compounds into class 1 and class 2 subsets and developed *k*NN QSAR LD_50_ models for each subset using the same protocol and the same cutoff criteria (*q*^2^ and *R*^2^ > 0.5) as for compounds in classes 1 and 2 that we generated by means of the moving regression. The purpose of this was to see if statistically significant QSAR models could be obtained for any random division of the original data into two classes. Independently, we applied the test to compounds in unique classes 1 and class 2 by randomizing their LD_50_ values and redeveloping training set models. Both Y-randomization tests were repeated 10 times.

## Results

### Failure of conventional QSAR modeling of rodent acute toxicity

The modeling set including 230 compounds with known rat LD_50_ data was partitioned into 32 training and test sets and the conventional *k*NN QSAR modeling approach was applied to all training sets as detailed in “Materials and Methods.” We characterized each training set model by its *q*^2^ value; for the five best training set models, these values ranged between 0.5 and 0.57. These five models were used for predicting LD_50_ values for the respective external validation set (23 compounds). However, for each of these models the *R*^2^ value for this external set was < 0.5. When we used other types of in-house or commercial QSAR methods (e.g., support vector machine or partial least square) and other types of descriptors (e.g., MolConnZ descriptors or molecular operating environment descriptors), we obtained no statistically significant predictive QSAR models (data not shown). Likewise, modeling of the mouse data set (211 modeling compounds and 24 external validation compounds) was unsuccessful (data not shown). This negative result corroborates the well-known inability of conventional QSAR modeling approaches to arrive at statistically significant and externally predictive models of *in vivo* toxicity.

### Data partitioning using the moving M-regression approach

**Figure 2:**
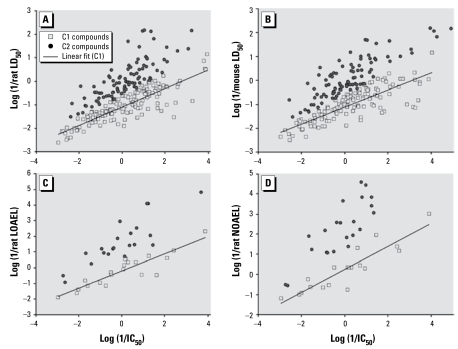
The identification of the baseline correlation between cytotoxicity (IC_50_) and various types of *in vivo* toxicity testing results. (*A*) Rat LD_50_. (*B*) Mouse LD_50_. (*C*) Rat LOAEL. (*D*) Rat NOAEL. C1, class 1; C2, class 2.

It is well known that *in vitro* cytotoxicity correlates poorly with *in vivo* toxicity end points when any relatively large set of compounds is considered. The ZEBET data set is no exception; cytotoxicity (IC_50_) correlates with acute toxicity (LD_50_) for only a fraction of the compounds in either the rat (Figure 2A) or mouse (Figure 2B) data sets. Most of the compounds are more toxic *in vivo* than *in vitro*. Similar patterns could be found between cytotoxicity and other *in vivo* toxicity end points, for example, rat chronic LOAEL and rat chronic NOAEL (Figures 2C, D).

To devise a mathematical means for identifying compounds with strong *in vitro*/*in vivo* correlation, we extended concepts that have been previously employed in calculating the “baseline regression” that correlated the aquatic toxicity of (some) chemicals with the logarithm of the *n*-octanol/water partition coefficient (log *P*) (Klopman et al. 1999, 2000; Mayer and Reichenberg 2006). Here, we have developed a novel approach, termed “moving M-regression,” to identify a subset of compounds with strong IC_50_ versus LD_50_ correlation. Using this method, we have partitioned compounds in the modeling set into two classes: class 1, compounds with acute toxicity that linearly correlate with cytotoxicity; and class 2, compounds with acute toxicity that do not correlate well with cytotoxicity, with these points positioned above the regression line.

This analysis for the rat ZEBET data set resulted in 122 compounds assigned to class 1, that is, within the linear regression correlation band between LD_50_ and IC_50_ values. The points corresponding to 93 out of 108 remaining compounds are located above the regression line band and are classified as class 2, whereas 15 compounds fall below the regression line (Figure 2A). Although these compounds are likely to be activity outliers, in the absence of an objective rationale for their outlier status, we merged them into class 1 to obtain the highest coverage of the resulting models and to provide a more realistic measure of external predictivity. Figure 2A and Equation 10 show the correlation between the LD_50_ and IC_50_ values of the resulting 137 class 1 compounds:


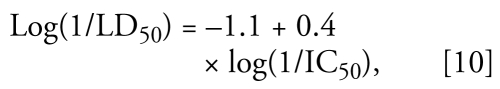


with *R*^2^ = 0.74, SE = 0.36, and *n* = 137. We also applied this approach to analyze the relationship between the *in vitro* IC_50_ and other *in vivo* toxicity data, including mouse LD_50_, rat chronic LOAEL, and rat chronic NOAEL (Table 1). The same trend was found for all data sets, that is, in all cases the data were partitioned into two classes: *a*) points on the baseline and *b*) points off the baseline (Table 1, Figure 2). We found the ratio of class 1 to class 2 compounds to be similar for each of the four *in vitro*/*in vivo* toxicity data sets. This result further supports the generality of the “moving M-regression” approach.

### Hierarchical QSAR modeling of the partitioned rodent toxicity data

Using class assignments from the data partitioning described above, we employed a two-step QSAR approach (Figure 1) for *a*) classification modeling (i.e., establishing that compounds assigned to classes 1 and 2 based on their biological activity data could be subdivided into the same classes based on their chemical structure), and *b*) predictive continuous modeling for all compounds in each class (i.e., estimation of the LD_50_ based on chemical structure, not IC_50_ data). For ZEBET rat data, we generated three modeling sets: set 1, 230 compounds (137 class 1 vs. 93 class 2) for classification modeling; set 2, 137 class 1 compounds; and set 3, 93 class 2 compounds for developing two continuous rat LD_50_ models. The analysis of these three data sets resulted in 252 classification models, as well as 1,207 continuous LD_50_ models for class 1 compounds and 40 continuous LD_50_ models for class 2 compounds that satisfied the statistical significance threshold criteria. Table 2 lists the statistical figures of merit for the best *k*NN models obtained for these three modeling sets.

To demonstrate that these QSAR models have significant external prediction accuracy, we have employed several concurrent approaches for model validation. First, following our general model validation work flow (Tropsha and Golbraikh 2007), we used 23 compounds excluded randomly from the entire data set as an external validation set. The following two-step prediction protocol for external compounds was used: *a*) *k*NN classification models were used to assign compounds to class 1 or class 2; and *b*) depending on the outcome, the respective class-specific continuous QSAR models was employed to predict the LD_50_ values for each compound. The results demonstrate that the overall accuracy of prediction for this external set is reasonably good. In the first step, the classification model had 65% prediction accuracy (the fraction of correctly identified class 1 and class 2 compounds). In the second step, we obtained *R*^2^ = 0.70, mean absolute error(MAE) = 0.39, and prediction coverage (i.e., the fraction of the external set compounds within the applicability domains of the models) of 74% for the external test set when combining the predictions for class 1 and class 2 compounds.

Second, we performed a 5-fold external CV analysis to test the robustness of the modeling outcome using 253 rat ZEBET compounds. The dataset was randomly split into five equal-size subsets of compounds and the modeling procedure was repeated five times, using each subset as a test set and the remaining four subsets as training set, as detailed in “Materials and Methods.” The statistical results of this exercise were as follows: slope_regr_ = 0.45 ± 0.01, *R*^2^_regr_ = 0.71 ± 0.04, *R*^2^_ext_ = 0.55 ± 0.05, MAE = 0.44 ± 0.04, coverage = 73 ± 3%.

Third, we performed Y-randomization tests to establish whether our models are statistically robust. Random partitioning of the compounds into two classes (10 times) produced only three (for class 1) and 28 (for class 2) models that satisfied the criteria of *q*^2^/*R*^2^ > 0.5, compared with 1,207 and 40, respectively, models for “moving M-regression”–assisted partitioning. Randomizing LD_50_ data generated no model with *q*^2^/*R*^2^ > 0.5 for class 1 and class 2 compounds.

Fourth, we performed additional Y-randomization analyses by randomly moving or rotating the correlation line (including negative correlation) and redefining compounds into classes 1 and 2. The randomly assigned class 1 and class 2 sets were used to develop QSAR LD_50_ models individually and the procedure was repeated 10 times. We found that at most, a very small number (< 7) of acceptable (*Q*^2^ > 0.5, *R*^2^ > 0.5) models could be developed.

Similar modeling results were obtained using the ZEBET mouse LD_50_ data. After partitioning 211 modeling set compounds into 119 class 1 compounds and 92 class 2 compounds, we developed 843 classification models for class 1 versus class 2, 236 continuous LD_50_ models for class 1 compounds, and 356 models for class 2 compounds. A two-step prediction protocol for evaluation of the 24 external compounds resulted in similarly good external prediction accuracy: *R*^2^ = 0.69, MAE = 0.42, and prediction coverage of 54%.

As a true external validation challenge, we have used our model to make predictions for the 115 compounds with rat LD_50_ data in the new ICCVAM data set. We compiled this data set after we finished the development of the above-described QSAR LD_50_ models, so it could be viewed as a true “blind” validation test. The statistical parameters of the prediction results for these compounds were *R*^2^ = 0.57, MAE = 0.48, and prediction coverage of 70%. Although somewhat less accurate than the results of the previous external prediction, this validation reinforces the statistical significance and utility of the model.

Y-randomization tests were also performed for the mouse LD_50_ data set. Similar to the rat data, after 10 random assignments of compounds into the two classes, we developed, at most, 4 (for class 1) and 38 (for class 2) models (*q*^2^/*R*^2^ > 0.5), compared with 843 and 236 models, respectively, when we used a classification model. Randomization of LD_50_ values produced no significant models.

### Stability of the in vitro and in vivo moving M-regression parameters

Because the regression correlation between *in vitro* (IC_50_) and *in vivo* (LD_50_) data is required to classify the modeling set compounds and, subsequently, to create the *k*NN classification models, this linear correlation is an essential factor to determine the robustness of our final models. Hence, the slope of the correlation and associated correlation coefficient (*R*^2^) should remain stable when new compounds are added into the modeling set. To validate this supposition, we compiled all available ZEBET and ICCVAM compounds with rat LD_50_ data to create a new modeling set, including the original modeling set (230 compounds), the external validation set (23 compounds), and additional data (115 compounds). We also included the compounds previously not used for modeling (inorganic, organometallic, and mixtures) because we used no chemical descriptors in this validation. Using the moving M-regression approach for all 425 compounds with IC_50_ and LD_50_ values, the resulting *in vitro*/*in vivo* correlation parameters are similar to those obtained from our original modeling set in Equation10:


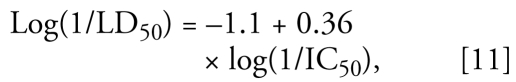


with *R*^2^ = 0.71, SE = 0.37, and *n* = 258. The proportions of class 1 and class 2 compounds and outliers among these 425 compounds were also comparable to those of the original modeling set of 230 compounds (Table 1). We conclude that adding new compounds into the modeling set, which should be important to improve the final model by enriching its chemical and biological diversity, does not affect the *in vitro*/*in vivo* regression statistics.

### Comparison between the two-step hierarchical LD_50_ QSAR model and TOPKAT

**Figure 3:**
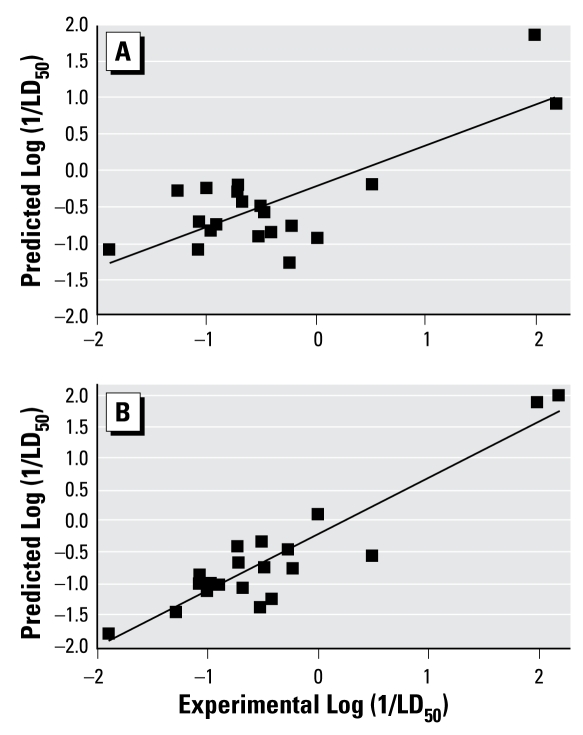
The correlation between experimental and predicted LD_50_ values for 27 external compounds within the applicability domain (*A*) using TOPKAT and (*B*) using the two-step model developed in this study.

We compared the performance of our modeling approach with that of TOPKAT software, version 6.1 (Accelrys 2009; Enslein 1988). Two types of comparison were considered. First, we have analyzed 27 of the 115 ICCVAM compounds that have been used neither for building our model nor in the TOPKAT LD_50_ training set. Figure 3 shows the correlation between the experimental and predicted LD_50_ values obtained from our model versus TOPKAT. The *R*^2^ and MAE of TOPKAT were 0.16 and 0.78, respectively, for all 27 compounds, which is considerably less than the same statistical parameters for prediction of the same data set using our model, *R*^2^ and MAE of 0.64 and 0.38, respectively. For seven compounds that were outside of the applicability domain for our model, the *R*^2^ and MAE using TOPKAT were 0.60 and 0.50, respectively, whereas our model produced values of 0.86 and 0.29, respectively (Table 3).

Second, we have used our models to predict acute toxicity compounds in the RTECS (Norager et al. 1978) data set (data were kindly provided by Todd Martin from the U.S. EPA), which contains approximately 7,000 compounds with rat LD_50_ data. We removed compounds that we found within the ZEBET data set, as well as inorganic compounds and mixtures. This procedure produced a library of 4,003 compounds spanning a diverse chemical space of organic molecules for which experimental rat LD_50_ data are available.

**Figure 4:**
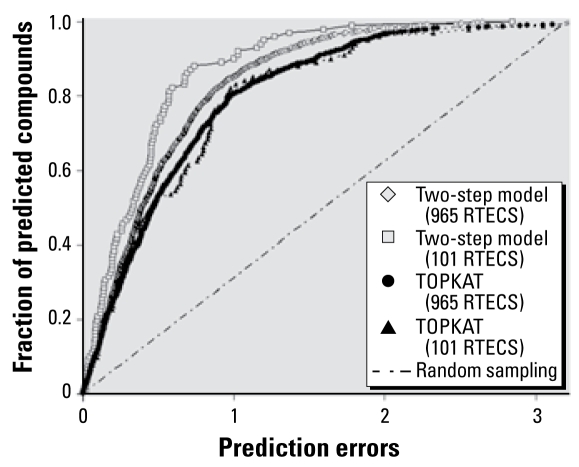
Fraction of compounds versus prediction errors obtained by the two-step rat LD_50_ model, TOPKAT, and random sampling for 965 and 101 RTECS compounds.

Because the size of the RTECS library is much larger than that of our original modeling set, we drew from our experience in using QSAR models for virtual screening (Oloff et al. 2005) and narrowed the model applicability domain. Consequently, predictions were made only for compounds that had greater than 70% confidence level in assigning them to either class 1 or class 2 in step 1 of our work flow (i.e., we required that > 70% of all QSAR models meeting our acceptability domain criteria would predict a compound in the same class). We determined that there were 1,562 compounds (out of 4,003) that were not included in the training set of TOPKAT rat LD_50_ model and for which predictions could be made based on the aforementioned criteria. The TOPKAT model predicted LD_50_ values for these compounds with an *R*^2^ = 0.16 and MAE = 0.78 (Figure 4, Table 3). The same parameters for the two-step QSAR model were 0.26 and 0.65, respectively. After implementing the applicability domain filter, we made predictions for 965 RTECS chemicals. TOPKAT model had parameters of *R*^2^ = 0.22 and MAE = 0.65; the same parameters for the two-step model were 0.33 and 0.54, respectively (Table 3), which is better than or comparable to prediction accuracy of various commercial QSAR modeling packages (Moore et al. 2003), albeit there is room for improvement.

It should be noted that the prediction accuracy of the two-step model can be improved by applying stricter criteria in the classification step. For instance, a 90% cutoff for correct class prediction results in prediction model statistics of *R*^2^ = 0.62 and MAE = 0.42, but the coverage of the model diminishes considerably to include 101 compounds (Table 3). The performance of TOPKAT for the same 101 compounds is poor: *R*^2^ = 0.26 and MAE = 0.66. Considering that the TOPKAT LD_50_ training set contains many more compounds (~ 6,000) than the training set used to develop the two-step model (~ 200), it is noteworthy that higher prediction accuracy can be achieved using our modeling approach for a much larger data set. Furthermore, our approach outperforms TOPKAT consistently over a range of error thresholds either for 965 RTECS compounds or for 101 RTECS compounds (Figure 4). In addition, we used the Wilcoxon test to calculate the *p*-values for the differences in MAEs obtained using two methods. Both for the whole set (965 compounds) and for the reduced set (101 compounds), the improvement achieved by our method, compared with TOPKAT, is statistically significant (*p* < 0.005).

One obvious reason that the prediction accuracy of our models for RTECS compounds is lower than that obtained from the external validation set of ICCVAM data is the difference in “activity” ranges of compounds in these two data sets. For example, the activity (log 1/activity, in millimolar units) of ZEBET compounds ranges from −2.61 to 2.30, whereas the activity range of RTECS compounds is considerably larger, from −3.34 to 4.21. It should be stressed that the *k*NN method used in our study cannot extrapolate in the activity space because external compound activity is predicted by averaging the activities of nearest-neighbor compounds in the training set as described in “Materials and Methods.” The MAE for the prediction of RTECS compounds that have experimental activity above 2 or below −2 is 1.14 log units. On the other hand, the MAE for the prediction for RTECS compounds that have experimental activity between −2 and 2 is considerably lower, 0.52 log units. The likely explanation for the better performance of our models in the latter range is that more than 90% of our modeling set compounds have rat LD_50_ activity in the same range, between −2 and 2. Increasing the diversity and activity range of compounds in the modeling set should significantly improve the prediction accuracy of our models.

## Discussion

The conventional wisdom in mechanistic and regulatory toxicology is that predictions of the *in vivo* toxicity end points from *in vitro* measures, even within the same species, are difficult. However, an approximate linear correlation between *in vitro* IC_50_ and rodent LD_50_, two of the widely acceptable benchmark parameters used for regulatory purposes, can be established for a significant fraction of the compounds. Indeed, we confirmed this notion by quantitative analysis of the IC_50_/LD_50_ relationships and devised an objective, computational means to partition compounds into two groups: those having good linear fit within a defined band, or those falling outside the band and exhibiting, for the most part, higher *in vivo* than *in vitro* toxicity. Our hypothesis to explain this observation is that, whereas cytotoxicity assays can reflect some of the toxicity mechanisms resulting in adverse health effects at the whole-animal level, the *in vitro* tests cannot fully reproduce the complex mechanisms of the *in vivo* toxicity. For example, it is well known that many compounds are not toxicants themselves but have metabolites that are toxic. We argue that the two-step prediction model based on chemical descriptors only that we developed in our studies also assists in identification of the compound subset that may act directly (i.e., without being biotransformed) and through mechanisms likely to be predictive of the potential *in vivo* effects. A similar argument was presented previously in ecotoxicity research where log *P* was found to be a mechanistically relevant predictor (Verhaar et al. 2000).

To further substantiate this argument, we considered the top 10 chemical fragment descriptors that were used most frequently in statistically significant QSAR models, that is, descriptors with the highest discriminatory power [see Supplemental Material, Table 3 (doi:10.1289/ehp.0800471.S1)]. It is noteworthy that the aromatic primary amine, “hydrazine,” and “sulfonamide” moieties, found within compounds that are known to be toxic both *in vitro* and *in vivo* (Alaejos et al. 2008; Carr et al. 1993; Toth 1988), were found predominantly in compounds of class 1. On the other hand, “pyrrolidine” and “aromatic tertiary amine” moieties, which require biotransformation (Domagala 1994), were predictors for class 2. We have also demonstrated that this objective division of the data set into two major groups affords robust hierarchical QSAR models, an assertion further supported by successive challenges to the models with external data sets, CV, and randomization of data.

The approach advocated in this study for biologically informed partitioning of structure–activity relationship data differs from conventional cheminformatics clustering approaches. Traditional methods partition compounds into multiple subgroups based on their chemical structure properties only (i.e., chemical descriptors). The underlying reasoning for chemically based clustering is that similar structures are expected to have similar biological properties and mechanisms of activity. However, it is a well-known limitation of structure–activity relationships that the absence or presence of a functional group or other minor change of the chemical structure may result in a large change of biological activity (Maggiora 2006). In our studies, the conventional chemical structure–based clustering method did not yield any statistically meaningful models, either global or local. The distribution of pairwise chemical similarities for all compounds within the modeling sets (class 1 vs. class 2) of rat LD_50_ values using DRAGON descriptors is very similar (data not shown). This observation reconfirms that chemical clustering would not have partitioned compounds in a way similar to the biological data-based partitioning.

## Conclusions

Although the cytotoxicity data generally show weak correlation with rodent acute toxicity, we have demonstrated that these data can be used to inform and improve QSAR modeling of *in vivo* acute toxicity. We have developed a novel two-step *k*NN QSAR modeling approach that affords a successful prediction of acute toxicity (LD_50_) values from chemical structure for both rats and mice. Furthermore, we predicted LD_50_ values for external compounds with accuracy, exceeding that of previously published QSAR models developed with the commercial (TOPKAT) software. It should be stressed that although *in vitro* cytotoxicity data have been used to establish the rules for partitioning most compounds into two classes, the ultimate models, both classification and continuous, employ chemical descriptors only. This vital feature of our approach makes it possible to achieve accurate predictions of rodent acute toxicity directly from chemical structure alone, even bypassing the need for *in vitro* studies of new compounds. We believe that this biological-data–based partitioning approach using *in vitro* toxicity data for the modeling set only, coupled with subsequent chemical-structure–based classification and continuous QSAR modeling techniques, holds promise for modeling other complex *in vivo* toxicity end points. This approach charts a future course for combining *in vitro* screening methods and QSAR modeling to prioritize chemicals for *in vivo* animal toxicity testing.

## Figures and Tables

**Table 1:** The results of data partitioning for the compounds with rat LD_50_, mouse LD_50_, rat chronic LOAEL, and rat chronic NOAEL data in ZEBET data set using cytotoxicity IC_50_ values.

Model	No. of C1 compounds	C1 ratio (%)	No. of C2 compounds	C2 ratio (%)
Rat LD_50_ (original set)	137	60	93	40
Mouse LD_50_	119	56	92	44
Rat LOAEL	21	49	21	51
Rat NOAEL	19	46	22	54
Rat LD_50_ (full data set)	258	61	167	39

Abbreviations: C1, Class 1; C2, Class 2.

**Table 2:** Statistical information for the five most statistically significant *k*NN QSAR models based on three modeling sets.

Model	N-training	Pred-training	N-test	Pred-test	NNN
The best *k*NN classification model for 137 class 1 versus 93 class 2 compounds					
1	173	0.84	55	0.73	1
2	147	0.86	74	0.70	1
3	193	0.83	37	0.73	1
4	165	0.86	59	0.70	1
5	173	0.81	55	0.75	1

The best *k*NN continuous model for 137 class 1 compounds					
1	103	0.66	34	0.81	3
2	103	0.73	34	0.71	2
3	111	0.71	26	0.74	3
4	115	0.65	22	0.79	5
5	77	0.73	60	0.71	2

The best *k*NN continuous model for 93 class 2 compounds					
1	80	0.61	13	0.84	2
2	77	0.67	16	0.77	1
3	80	0.69	13	0.74	1
4	80	0.65	13	0.76	2
5	79	0.63	14	0.78	2

Abbreviations: NNN; number of the nearest neighbors used for prediction; N-test, number of compounds in the test set; N-training, number of compounds in the training set; Pred-test, the overall predictivity of the test set (correct classification rate for classification models, *R*^2^ for continuous models); Pred-training, the overall predictivity of the training set (correct classification rate for classification models, *q*^2^ for continuous models).

**Table 3:** Comparison between TOPKAT and the two-step model prediction of the external compounds.

	Two-step model		TOPKAT	
Measure	No applicability domain	With applicability domain	No applicability domain	With applicability domain
Prediction of 27 new ZEBET compounds				
*R*^2^	0.64	0.86	0.16	0.60
MAE	0.38	0.29	0.78	0.50
Coverage (%)	100	67	100	67

Prediction of 1,562 RTECS compounds with 70% confidence level				
*R*^2^	0.26	0.33	0.19	0.22
MAE	0.65	0.54	0.76	0.65
Coverage (%)	100	62	100	62

Prediction of 1,562 RTECS compounds with 90% confidence level				
*R*^2^	0.42	0.62	0.19	0.26
MAE	0.60	0.42	0.84	0.66
Coverage (%)	12	6	12	6
